# Adipose-derived stromal cells modulating composite allotransplant survival is correlated with B cell regulation in a rodent hind-limb allotransplantation model

**DOI:** 10.1186/s13287-020-01961-8

**Published:** 2020-11-11

**Authors:** Chien-Chang Chen, Rong-Fu Chen, Jheng-Syuan Shao, Yun-Ting Li, Yu-Chi Wang, Gerald Brandacher, Jiin-Haur Chuang, Yur-Ren Kuo

**Affiliations:** 1 Division of Plastic & Reconstructive Surgery, Department of Surgery, Kaohsiung Medical University Hospital, 100 Tzyou 1st Rd., Kaohsiung, 80756 Taiwan; 2 Graduate Institute of Clinical Medical Sciences, College of Medicine, Chang Gung University, Kaohsiung, Taiwan; 3 VCA Center, Department of Plastic & Reconstructive Surgery, Johns Hopkins School of Medicine, Baltimore, MD USA; 4 Department of Surgery, Kaohsiung Chang Gung Memorial Hospital and Chang Gung University College of Medicine, Kaohsiung, Taiwan; 5 Faculty of Medicine, College of Medicine, Orthopaedic Research Center, Regenerative Medicine and Cell Therapy Research Center, Kaohsiung Medical University, Kaohsiung, Taiwan; 6 Department of Biological Sciences, National Sun Yat-sen University, Kaohsiung, Taiwan; 7 Academic Clinical Programme for Musculoskeletal Sciences, Duke-NUS Graduate Medical School, Singapore, Singapore

**Keywords:** Adipose-derived stromal cells, Regulatory B cell, Vascularized composite allotransplantation

## Abstract

**Background:**

Our previous studies demonstrated that adipose-derived mesenchymal stromal cells (ASCs) have immunomodulatory effects that prolong allograft survival in a rodent hind-limb allotransplant model. In this study, we investigated whether the effects of immunomodulation by ASCs on allograft survival are correlated with B cell regulation.

**Methods:**

B cells isolated from splenocytes were cocultured with ASCs harvested from adipose tissue from rodent groin areas for in vitro experiments. In an in vivo study, hind-limb allotransplantation from Brown-Norway to Lewis rats was performed, and rats were treated with ASCs combined with short-term treatment with anti-lymphocyte serum (ALS)/cyclosporine (CsA) as immunosuppressants. Peripheral blood and transplanted tissue were collected for further analysis.

**Result:**

An in vitro study revealed that ASCs significantly suppressed lipopolysaccharide-activated B cell proliferation and increased the percentage of Bregs. The levels of immunoregulatory cytokines, such as TGF-β1 and IL-10, were significantly increased in supernatants of stimulated B cells cocultured with ASCs. The in vivo study showed that treatment with ASCs combined with short-term ALS/CsA significantly reduced the B cell population in alloskin tissue, increased the proportion of circulating CD45Ra^+^/Foxp3^+^ B cells, and decreased C4d expression in alloskin.

**Conclusion:**

ASCs combined with short-term immunosuppressant treatment prolong allograft survival and are correlated with B cell regulation, C4d expression and the modulation of immunoregulatory cytokines.

## Introduction

Vascularized composite allotransplantation (VCA) has been used successfully in clinical applications in areas such as the face, upper limbs, larynx, penis, and uterus. However, VCAs are not routinely performed because a vast majority of VCA recipients develop chronic rejection and consequently require lifelong administration of immunosuppressive agents, which have adverse side effects. Therefore, the development of new strategies to prevent the lifelong use of immunosuppressants or the induction of immune tolerance is urgently needed [1–4].

Mesenchymal stromal cells (MSCs) are multipotent stromal cells present in various tissues, such as bone marrow, umbilical cord, and adipose tissue, that are capable of differentiating into various mesenchymal tissues [5]. MSCs are reported to possess a variety of immunomodulatory properties that are mediated through cell-to-cell contact or secretion of different soluble factors; thus, MSCs have emerged as potent cell-based therapeutics to treat autoimmune diseases and induce immune tolerance in allotransplantation [6, 7]. Our former study revealed that combined short-term anti-lymphocyte serum and cyclosporine A (ALS/CsA) treatment group could prolong allotransplant survival but still early rejection in a rodent hind-limb allotransplantation model [8]. However, adipose-derived stromal cells (ASCs) combined short-term ALS/CsA could significantly increase the allotransplant survival as compared to that in ALS/CsA alone group. These indicate ASCs play an important role to modulate allograft survival. Our previous studies demonstrated that ASCs combined with short-term immunosuppressants (ALS/CsA) prolonged allograft survival through suppression of T cell proliferation and activation of regulatory T cell populations in a rodent/swine hind-limb VCA model [8–10].

However, in addition to T cell participation in the transplantation rejection process, the study revealed that B cells also play an important role through the production of antibodies against allografts and the secretion of pro-inflammatory cytokines [11]. Moreover, recent studies have also shown that a subset of B cells called regulatory B cells (Bregs) could regulate immune responses and maintain immune tolerance through the production of immunomodulatory cytokines, such as interleukin-10 (IL-10) and transforming growth factor (TGF)-β1 [12, 13]. Thus, the modulation of B cells may be a promising strategy to induce immune tolerance during transplantation. MSCs are reported to regulate B cell proliferation and induce the activation of the Breg population [14–19]. However, the immunomodulatory effects of ASCs on B cells during composite tissue allotransplantation remain unclear.

Complement components are important factors involved in innate immunity, and it has been established that they play a crucial role in the rejection of transplanted allografts [20]. One of the complement split products, C4d, can be used as a marker for antibody-mediated rejection in allotransplants [21].

In this study, we investigated whether ASCs could regulate B cell proliferation and modulate Breg induction and cytokine production in vitro. To further examine the immunomodulatory effects of ASCs on B cells in vivo, animals with rodent hind-limb allotransplantation were treated with ASCs combined with short-term immunosuppressants. Analysis of B cell proliferation and the induction of the Breg population and the detection of the deposition of complement C4d were conducted.

## Materials and methods

### Animals

Eighteen male Lewis (LEW) rats aged 8 to 12 weeks and weighing 250 to 350 g were used as recipients. Eighteen Brown-Norway (BN) rats with similar ages and weight ranges were used as donors. All animals were purchased from the National Laboratory Animal Breeding and Research Center in Taipei, Taiwan. The experiments were approved by the Institutional Animal Care and Use Committee at Kaohsiung Medical University, Taiwan.

### Isolation and culture of adipose-derived stromal cells

Adipose-derived stromal cell (ASC) isolation was performed according to a previously described method [22]. Groin adipose tissues from six Lewis rats were minced, washed, and digested with 0.4% collagenase type I for 30 min at 37 °C. The suspension was centrifuged at 500×*g* for 5 min at room temperature, and the red blood cells were lysed with RBC lysis buffer. The cells were then washed with phosphate-buffered saline, resuspended in maintenance medium, and seeded in culture dishes for 24 h. Next, the supernatant and nonadherent cells were discarded, and the attached cells were considered as passage 0 of the ASCs. ASCs were cultured in maintenance medium consisting of Dulbecco’s modified Eagle medium supplemented with 10% fetal bovine serum and 1% penicillin-streptomycin at 37 °C in a humidified atmosphere with 5% carbon dioxide. To prevent spontaneous differentiation, cultures were maintained at sub-confluent levels (< 80% confluency). On average 2 × 10^6^ cells were plated into a Falcon™ T75 tissue culture flask and usually passaged cells at a ratio of 1:3. Passaging of ASC cultures was performed after removal of medium, washing with PBS, and digestion with 2.5% trypsin solution in PBS supplemented with 0.23 mM ethylenediaminetetraacetic acid (EDTA). Passaged cultures were defined as passage 1. The ASCs from passages 3 and 6 were used for experiments.

ASCs were cultured in maintenance medium consisting of Dulbecco’s modified Eagle medium supplemented with 10% fetal bovine serum (HyClone) and 1x antibiotic-antimycotic solution (Gibco) at 37 °C in a humidified atmosphere with 5% carbon dioxide. The ASCs were characterized by flow cytometry according to positive surface staining for CD29, CD44, CD73, CD90, MHC-I, and CD106 and negative staining for CD31, CD34, CD45, and MHC-II. The capacity of ASCs to differentiate into adipocytes, osteoblasts, and chondrocytes was tested by following our previously described procedures [9].

### Isolation and culture of B cells

Splenocytes were labeled magnetically with anti-rat kappa microbeads (Miltenyi Biotech, Inc., Auburn, CA) and then transferred onto a column placed in a magnetic field by following the manufacturer’s instructions. The magnetically labeled Igk^+^ B cells that were retained on the column were eluted to positively select for Igk^+^ B cells. The isolated B cells were examined by flow cytometry for positive staining with CD45Ra and consisted of at least 95% pure CD45Ra^+^ B cells. The B cells were maintained in Roswell Park Memorial Institute 1640 medium supplemented with 10% fetal bovine serum, 2 mM glutamine, 1 mM sodium pyruvate, 1x MEM NEAA (Gibco), 55 μM 2-mercaptoethanol, and 1x antibiotic-antimycotic solution (Gibco) at 37 °C in a humidified atmosphere with 5% carbon dioxide.

### Bromodeoxyuridine (BrdU) cell proliferation assay

ASCs were seeded in 96-well plates as stimulator cells and were inactivated by pretreating them with mitomycin C (Sigma, 10 μg/mL) for 1 h at 37 °C. The B cells were isolated and added to the ASCs at a B cell: ASC ratio of 5:1 and incubated for 3 days. In the lipopolysaccharide (LPS, Sigma, 100 μg/ml) treatment groups, LPS was added to the culture medium for the last 2 days of culture. A BrdU cell proliferation assay kit (Millipore, Billerica, MA) was used to evaluate B cell proliferation. Absorbance was measured at 450 and 540 nm in an enzyme-linked immunosorbent assay microplate reader.

### Coculture of ASCs and B cells

ASCs were seeded in 24-well plates and pretreated with mitomycin C (Sigma, 10 μg/mL) for 1 h at 37 °C. B cells were isolated and added to the ASCs at a B cell: ASC ratio of 5:1 in DMEM medium and incubated for 3 days. LPS was added to the culture medium for the last 2 days. Then, the cells were collected and washed with PBS twice for flow cytometric analysis.

### Flow cytometry assessment of CD45Ra^+^/Foxp3^+^ B cells

The cells were incubated with 5 μl of monoclonal antibodies against CD45Ra (PE, BD Biosciences) for 30 min at room temperature to identify the B lymphocytes. These were incubated with 5 μl anti-Foxp3 for 90 min at 4 °C to identify the regulatory B cells (Perk; eBioscience, Inc.). After incubation, the cells were centrifuged, washed, and analyzed on a BD Accuri C6 flow cytometer (BD Biosciences Pharmingen). Data were analyzed using BD Accuri C6 software. Samples were analyzed at three technical replicates in each of the three independent experiments. The cells were collected again and counted after the coculture of ASCs and B cells.

### Detection of IL-10, TGF-β1, IFN-γ, and IL-17F cytokines

The concentrations of IL-10, TGF-β1, interferon-γ (IFN-γ), and interleukin-17F (IL-17F) in the coculture medium were determined by an enzyme-linked immunosorbent assay (ELISA) kit (R&D Systems, Minneapolis, MN) by following the manufacturer’s instructions.

### Orthotopic hind-limb allotransplantation model

The rodent hind-limb allotransplant model was generated according to our previous studies [8, 23, 24]. Under general anesthesia with isoflurane, the hind limbs of donor BN rats were amputated at the mid-thigh level and placed in gauze soaked with cold normal saline; the hind limbs of recipient LEW rats were amputated at the same level following the preparation of the recipients’ vessels. Osteosynthesis of the donor and recipient femurs was performed by using an 18-gauge needle as the intramedullary rod. The ventral and dorsal muscle groups were repaired using 4–0 interrupted silk sutures. The femoral vein and artery were anastomosed with 10–0 nylon sutures with the aid of a stereomicroscope (Zeiss, Gottingen, Germany). The skin was closed with 4–0 silk sutures.

### Animal experimental design

**Fig. 1:**
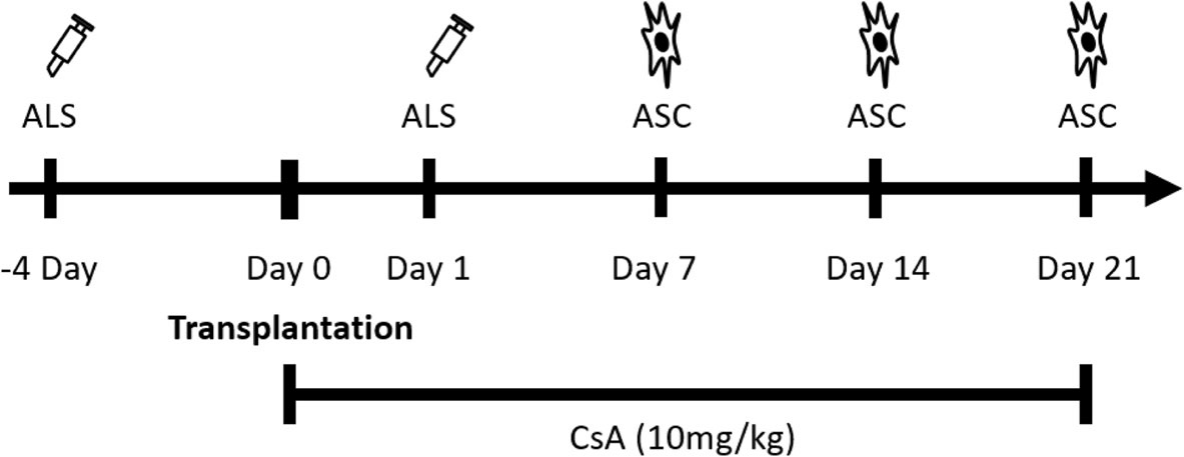
Flowchart showing the treatment protocol. Orthotopic hind-limb allotransplants were performed between donor Brown-Norway (BN) and recipient Lewis (LEW) rats (day 0) (Each subgroup *n* = 6 rats). Rats in the ASC/ALS/CsA treatment group received CsA (days 0 to 21), ALS (day − 4 and day 1), and ASCs (2 × 10^6^ cells/treatment administered intravenously on days 7, 14, and 21). The dosage and protocols mimicked those used in our previous study. ASC, adipose-derived stromal cell; ALS, anti-lymphocyte serum; CsA, cyclosporine A

Orthotopic hind-limb allotransplantation from BN to LEW was performed on day 0 (*N* = 12, each subgroup *n* = 6). Group 1 received no treatment and functioned as a control. Rats in the ASC/ALS/CsA treatment group (Group 2) were treated with a combination of CsA at 10 mg/kg per day from days 0 to 21, ALS (0.5 ml, intraperitoneally administered 4 days before and 1 day after transplantation), and ASCs (2 × 10^6^ cells/treatment on days 7, 14, and 21 after transplantation) (Fig. 1). The dosages of the ASCs and the treatment protocol were following our previously established model [8, 23, 24]. Serum and alloskin biopsy samples were obtained from recipients at 2 weeks and specific week post-transplantation (control and ASC/ALS/CsA treatment group).

### Immunohistochemical staining

Tissue sections were subjected to immunohistochemical staining of CD45Ra^+^ B cells and C4d to investigate the numbers of CD45Ra^+^ B cells and the expression of C4d in tissues after transplantation on specified days. A horseradish peroxidase-diaminobenzidine kit was used (BioGenex Laboratories, Inc., San Ramon, CA) for all IHC staining experiments. After endogenous peroxidase activity was quenched with 3% hydrogen peroxide for 10 min at room temperature, the tissue sections were stained with anti-CD45Ra or anti-C4d antibodies at a 1:50 dilution in phosphate-buffered saline. The tissue sections were incubated with antibodies at 4 °C in the dark overnight and were then incubated with biotinylated anti-mouse secondary antibody for 30 min. Visualization of specific binding was performed by enzymatic conversion of the chromogenic substrate 3,3′-diaminobenzidine into a brown precipitate by horseradish peroxidase. After counterstaining with hematoxylin, the sections were mounted, cleaned, and cover-slipped.

### Statistical analysis

Data are expressed as the mean ± standard error. Significant differences between experimental groups were analyzed using the *t* test. Log transformation was performed before statistical analysis to confirm normality and equal variances for flow cytometric data. Results are expressed as means ± SD. A value of *p* < 0.05 was considered statistically significant.

## Results

### ASCs suppressed B cell proliferation and increased the regulatory B cell population in vitro

**Fig. 2:**
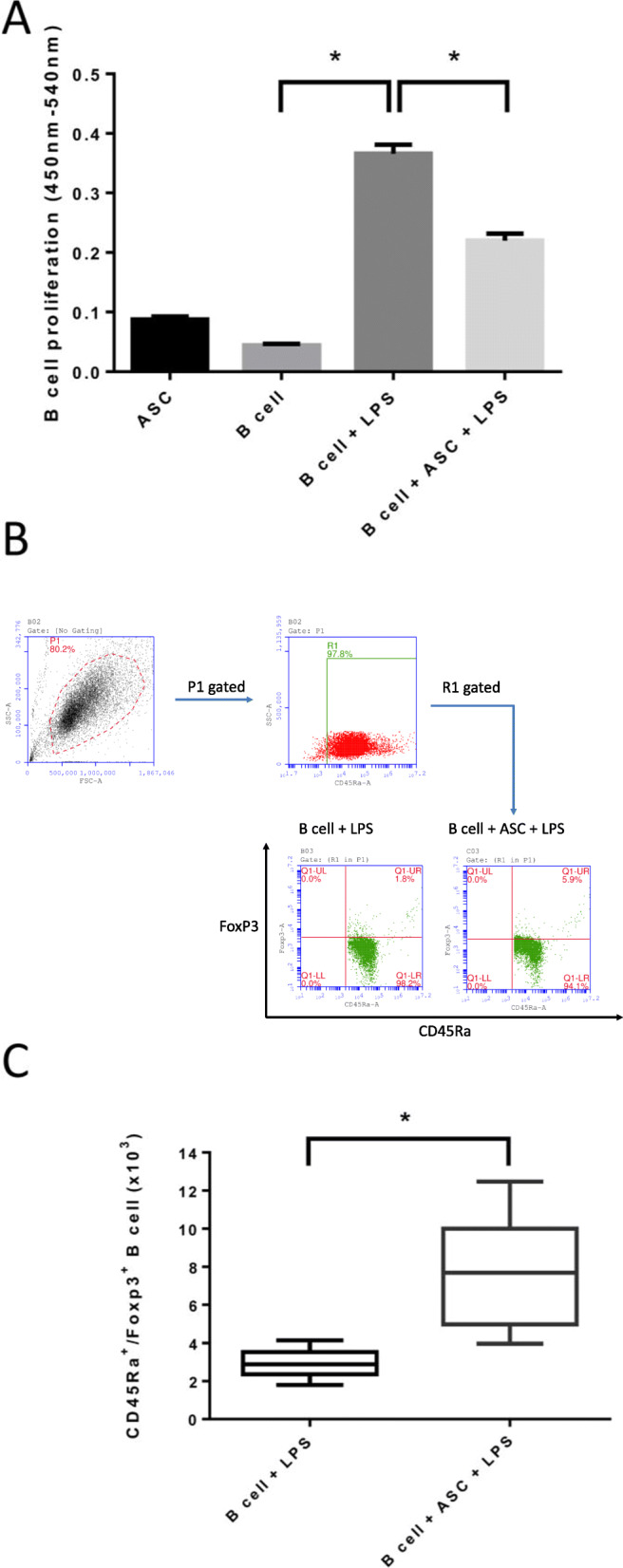
B cell proliferation assay and flow cytometric analysis of CD45Ra^+^/Foxp3^+^ regulatory B cells in vitro. **a** Bromodeoxyuridine cell proliferation assays showed a decrease in the proliferation of LPS-stimulated B cells when these cells were cocultured with ASCs. *P < 0.05. **b** Dot plots gated on lymphocyte (P1 region) and CD45Ra^+^ B cells (R1 region). Flow cytometric analysis revealed a significant increase in the proportion of the CD45Ra^+^/Foxp3^+^ regulatory B cell subset when these cells were cocultured with ASCs compared to that in the B cells alone group. **c** The number of CD45Ra^+^/Foxp3^+^ regulatory B cells were increased among B cells cocultured with ASCs compared to that in controls. Each experiment repeated at least three times. *P < 0.05

To investigate the immunomodulatory effects of ASCs on B cells, B cells were cocultured with ASCs in vitro. BrdU assays showed that B cell proliferation was increased in the presence of LPS activation (B cell + LPS group) when compared to that in the B cell alone group (Fig. 2a). However, the effects on cell proliferation of LPS-activated B cells were significantly suppressed when the B cells were cocultured with ASCs. Moreover, flow cytometric analysis revealed that the proportions of CD45Ra^+^/Foxp3^+^ regulatory B cell subsets among activated B cells cocultured with ASCs were significantly increased compared to those in the B cell activation group (Fig. 2b). The number of CD45Ra^+^/Foxp3^+^ B cells was also increased in the ASC-B cell coculture group (Fig. 2c). These data suggest that ASCs have immunoregulatory effects that decrease B cell proliferation and increase the proportion of regulatory B cells.

### ASCs induced the expressions of immunoregulatory cytokines in vitro

**Fig. 3:**
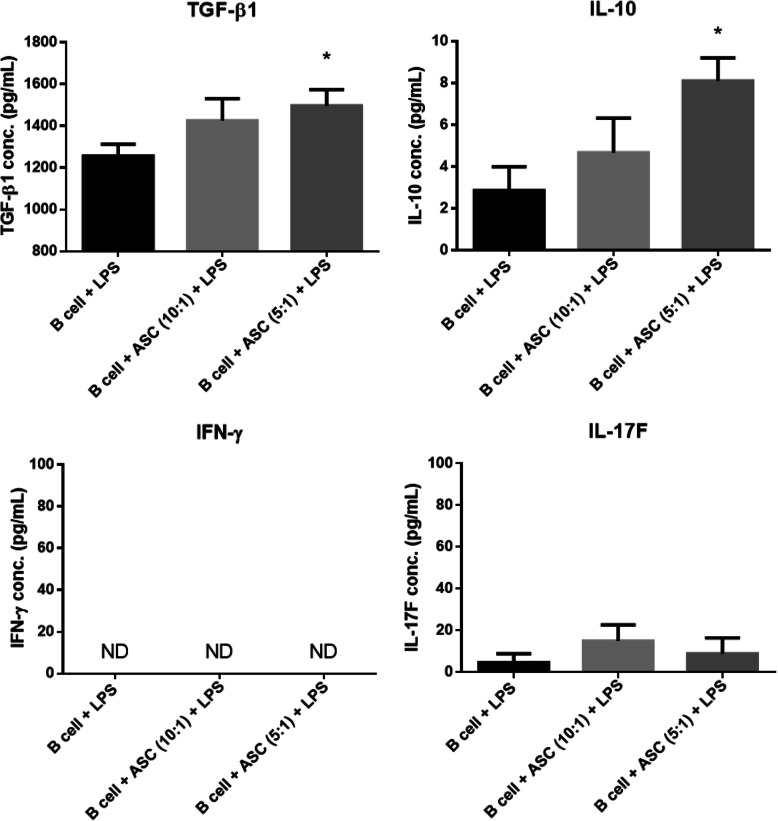
Expression of TGF-β1, IL-10, IFN-γ, and IF-17F in culture medium. The concentrations of TGF-β1 and IL-10 were significantly increased in B cells cocultured with ASCs. However, the IFN-γ level was not detected in any treatment group. IL-17F production was not changed by B cell/ASC coculture compared to that in the LPS-stimulated B cell alone group. All experiments repeated at least three times. *P < 0.05. ASC, adipose-derived stromal cell; LPS, lipopolysaccharide; ND, not detected

To examine the production of anti- and pro-inflammatory cytokines in the coculture medium, TGF-β1, IL-10, IFN-γ, and IL-17F expressions in the culture medium were determined by ELISA. The concentrations of TGF-β1 and IL-10 were significantly increased in LPS-stimulated B cells cocultured with ASCs. In contrast, IFN-γ was not detected in any of the study groups. The concentration of IL-17F was not significantly changed in the stimulated B cell and ASC coculture group compared to that in the stimulated B cell alone group (Fig. 3). These results indicate that ASC modulation of B cell activation is involved in the secretion of anti-inflammatory cytokines, such as TGF-β1 and IL-10.

### ASCs prolonged allotransplant survival and were correlated with the modulation of the B cell population in vivo

**Fig. 4:**
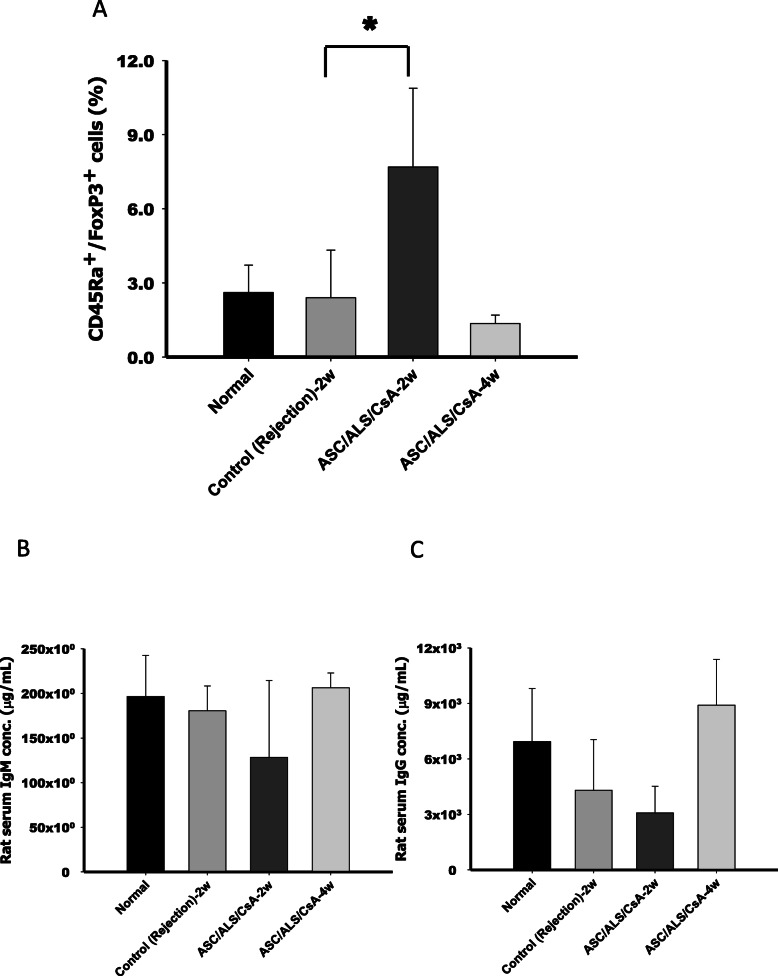
Circulating CD45Ra^+^/Foxp3^+^ regulatory B cells and circulating IgM/IgG levels in orthotopic hind-limb allotransplants. Flow cytometric analysis revealed a significant increase in CD45Ra^+^/Foxp3^+^ regulatory B cells in the ASC/ALS/CsA group at 2 weeks after transplantation compared with that in the controls (**a**). The procedure was repeated at least three times. Enzyme-linked immunosorbent assay analysis of recipient peripheral blood serum at 2 and 4 weeks post-transplantation revealed that the concentrations of IgM (**b**) and IgG (**c**) have a trend of decrease in ASC/ALS/CsA at 2 weeks post-transplantation compared to those in the control group. ASC, adipose-derived stromal cell; ALS, anti-lymphocyte serum; CsA, cyclosporine A. *P < 0.05

**Fig. 5:**
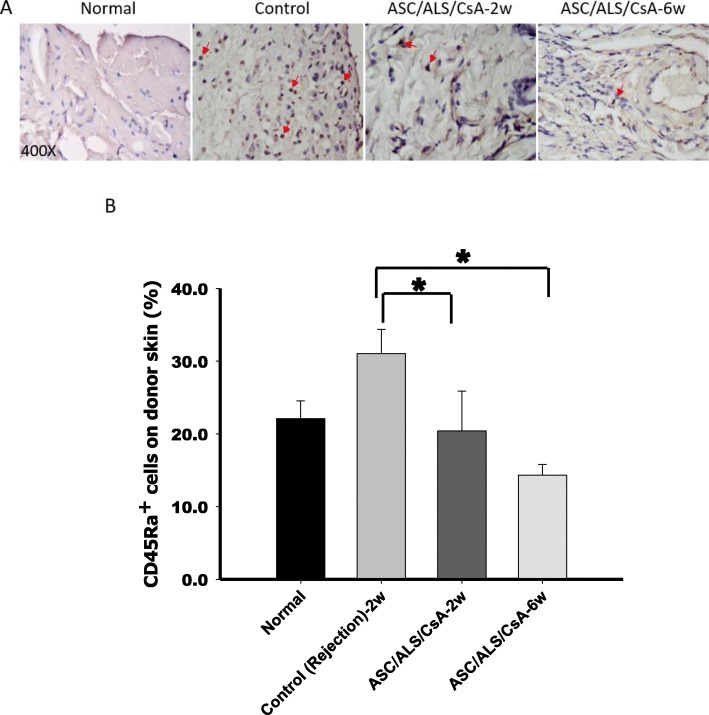
Expression of CD45Ra^+^ B cells in alloskin tissue. **a** Immunohistochemical staining of CD45Ra^+^ B cells in alloskin biopsies obtained at 2 and 6 weeks post-transplantation. Tissue sections obtained from six specimens were analyzed. Four randomly selected areas were then photographed at × 400 magnification. Images were captured and analyzed with Image-Pro Plus image analysis software. **b** IHC revealed a significant decrease n CD45Ra^+^ B cells in alloskin tissues in the ASC/ALS/CsA-treated group when compared with that in the control (rejection) group at 2 weeks post-transplantation. *P < 0.05

Our former results demonstrated that ASCs prolonged allograft survival in a rodent hind-limb allotransplant model [8]. To evaluate the immunomodulatory capacities of B cells in vivo, flow cytometric analysis of recipient peripheral blood was performed. The results revealed that the population of CD45Ra^+^/Foxp3^+^ regulatory B cells was significantly increased in the ASC/ALS/CsA treatment group 2 weeks after hind-limb allotransplantation compared to that in controls (Fig. 4a) and was skewed toward normal at 4 weeks after transplantation. ELISA assays of circulating IgM and IgG were detected from recipient peripheral blood. The concentration of circulating IgM and IgG revealed a trend of decease in the ASC/ALS/CsA treatment group at 2 weeks post-transplantation, consistent with the increase in the percentage of the regulatory B cell population in blood, and were restored at week 4 after transplantation (Fig. 4b, c). These data suggest that ASCs could modulate early IgM and IgG production of B cells at 2-weeks post-transplantation. The IHC staining of CD45Ra^+^ cells in alloskin tissue was decreased in the group treated with ASCs and short-term immunosuppressants (ALS/CsA) in the second and sixth weeks after transplantation compared to that in the controls (Fig. 5a, b). These data indicated that ASC treatments could modulate B cells by increasing the proportion of the regulatory B cell subset.

### ASCs reduced C4d expression in transplanted tissue

**Fig. 6:**
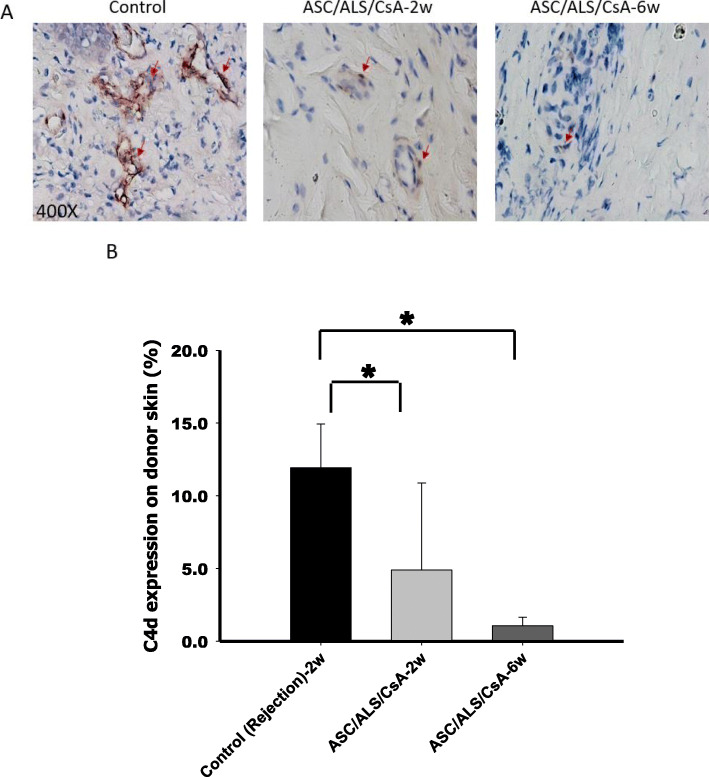
IHC staining of C4d in transplanted tissue. **a** Immunohistochemical staining of C4d in alloskin biopsy specimens obtained at 2 and 6 weeks post-transplantation. Tissue sections obtained from six specimens were analyzed. **b** IHC staining analysis revealed a decrease in the expression of C4d in the ASC/ALS/CsA-treated group at 2nd and 6th weeks compared with that in the control (rejection) group at 2 weeks post-transplantation. *P < 0.05

C4d, as a marker of complement activation, has been used as an indicator for the evaluation of cellular- and antibody-mediated rejection in a variety of tissues. The IHC staining analysis of C4d expression in transplanted alloskin tissue showed a significant reduction in C4d expression in the ASC/ALS/CsA treatment group at 2 and 6 weeks after transplantation compared to that in the control group (Fig. 6a, b). These results indicate that ASCs could decrease cellular- and antibody-mediated reactions and ameliorate allograft rejection.

## Discussion

The immunomodulatory effects of MSCs make them a promising tool for the treatment of immune diseases and organ transplantations. Our previous studies have demonstrated that the optimal dosage of ASCs combined with short-term immunosuppressants could prolong allotransplant survival and ameliorate transplant rejection. One of the main mechanisms is associated with the modulation of T cell proliferation, induction of the Treg population, and production of immunoregulatory cytokines, resulting in immune tolerance [8, 10, 25].

However, B cells also contribute to transplantation rejection through their ability to present antigens, secrete pro-inflammatory cytokines, and produce antibodies [20, 21, 26]. The production of donor-specific antibodies (DSAs) against donor human leukocyte antigen (HLA) is the primary cause of developing antibody-mediated rejection (AMR) [20, 26]. After transplantation, DSAs target donor endothelial cells, and then trigger immune responses and classical complement cascade which causes the deposition of C4d, leading to allograft damage and loss [20]. Previous studies have showed that mesenchymal stem cells have the ability to modulate the proliferation of B cells, population of Breg and the production of antibodies and cytokines [14, 15]. Recent studies have highlighted the existence of B cells with regulatory properties, which have been termed Bregs and are analogous to regulatory T cells (Tregs). Bregs have been found to play a role in autoimmune disease, malignancies, and infections and may also be a factor in solid organ transplantation [11]. Their regulatory function may be directly accomplished by the production of the regulatory cytokines IL-10 and TGF-β and/or by the ability of B cells to interact with pathogenic T cells to inhibit harmful immune responses. Previous studies have shown that MSCs can modulate B cell regulation and cytokine production [14–19]. However, it remains unclear whether MSCs can regulate B cell function during composite tissue allotransplantation.

In this study, we investigated the immunomodulatory effects of ASCs on B cells in vitro. Our data revealed that the proliferation of activated B cells was suppressed by ASCs. In contrast, ASCs induced an increase in CD45Ra^+^/Foxp3^+^ expression in B cells compared to that in B cells that were not cocultured with ASCs. Besides, an ELISA assay showed that the concentrations of IL-10 and TGF-β1 in culture medium were significantly increased in the presence of B cells cocultured with ASCs when compared to that in the presence of B cells alone [12, 13]. However, IFN-γ and IL-17F were not affected by coculture with ASCs. This indicated that modulation of B cell activation and enhancement of Breg expression by ASCs is correlated with the production of anti-inflammatory cytokines but not pro-inflammatory cytokines. In this study, it is hard to verify what cells produced the cytokines. Further study will be interesting to separate ASCs and B cells and verify what cells produced the cytokines in the coculture system.

In our in vivo study, a rodent hind-limb allotransplantation was used as a VCA model. Our previous study showed that a regimen consisting of ASCs combined with short-term ALS/CsA treatment (ASC/ALS/CsA group) significantly prolonged allograft survival when compared to the regimens used in the control-rejection and ALS/CsA groups [8]. Studies have also revealed that alloskin tissue contains the most potent antigens found in organ tissue [27]. In this study, IHC staining showed that an enormous number of CD45Ra^+^ B cells accumulated in alloskin 2 weeks after transplantation compared with that in the untreated control group. However, the number of CD45Ra^+^ B cells was significantly decreased in transplanted tissue in the combined ASC/CsA/ALS group. These results are consistent with our in vitro data showing that ASCs suppressed B cell proliferation. Moreover, flow cytometric analysis of recipient peripheral blood revealed that the percentage of CD45Ra^+^/Foxp3^+^ B cells was significantly increased in the combined ASC/ALS/CsA group at 2 weeks post-transplantation. The percentage of Bregs was skewed toward normal at week 4 after transplantation. In contrast, the concentration of IgM and IgG expressions in recipient circulating blood revealed a trend of decrease at 2 weeks after transplantation in the ASCs/ALS/CsA treatment group, consistent with increasing the percentage of CD45Ra+/Foxp3+ B cells expression in the circulating blood, and were restored toward normal at 4 weeks after transplantation. These findings indicate that ASCs could attenuate the immune response of B cells to allografts and modulate B cell subsets.

In the post solid organ transplantation context, B cells mediate humoral rejection by producing HLAs and DSAs while also providing costimulatory signals to T cells. The complement system, a component of the innate immune system [26], has also been demonstrated to modulate adaptive immunity and bridge the innate and adaptive immune responses. Studies have revealed that DSAs target donor endothelial cells and then trigger immune responses and the classical complement cascade, which causes deposition of C4d and leads to allograft damage and rejection after transplantation [20, 21, 26]. Thus, C4d is a common rejection marker related to B cells. Studies have shown that MSCs can reduce antibody production by B cells [28–30]. Thus, our data indicate that the treatment of ASCs could inhibit antibody production in the rodent hind-limb allotransplantation model. In this study, the expression of C4d was significantly decreased in transplanted tissue in the ASCs/ALS/CsA treatment group compared to that in the control. We found that the decrease in C4d deposition was correlated with the decrease in the B cell population in the transplanted tissue. This indicates that ASCs reduced B cell proliferation and decreased the deposition of C4d, which suppressed rejection. However, in this study, we could not find direct evidence to determine the signaling pathway by which ASCs suppress C4d expression. Further studies are needed to elucidate the biomechanisms involved in how ASCs suppress the activation of the complement-related pathway by regulating B cells.

## Conclusion

**Fig. 7:**
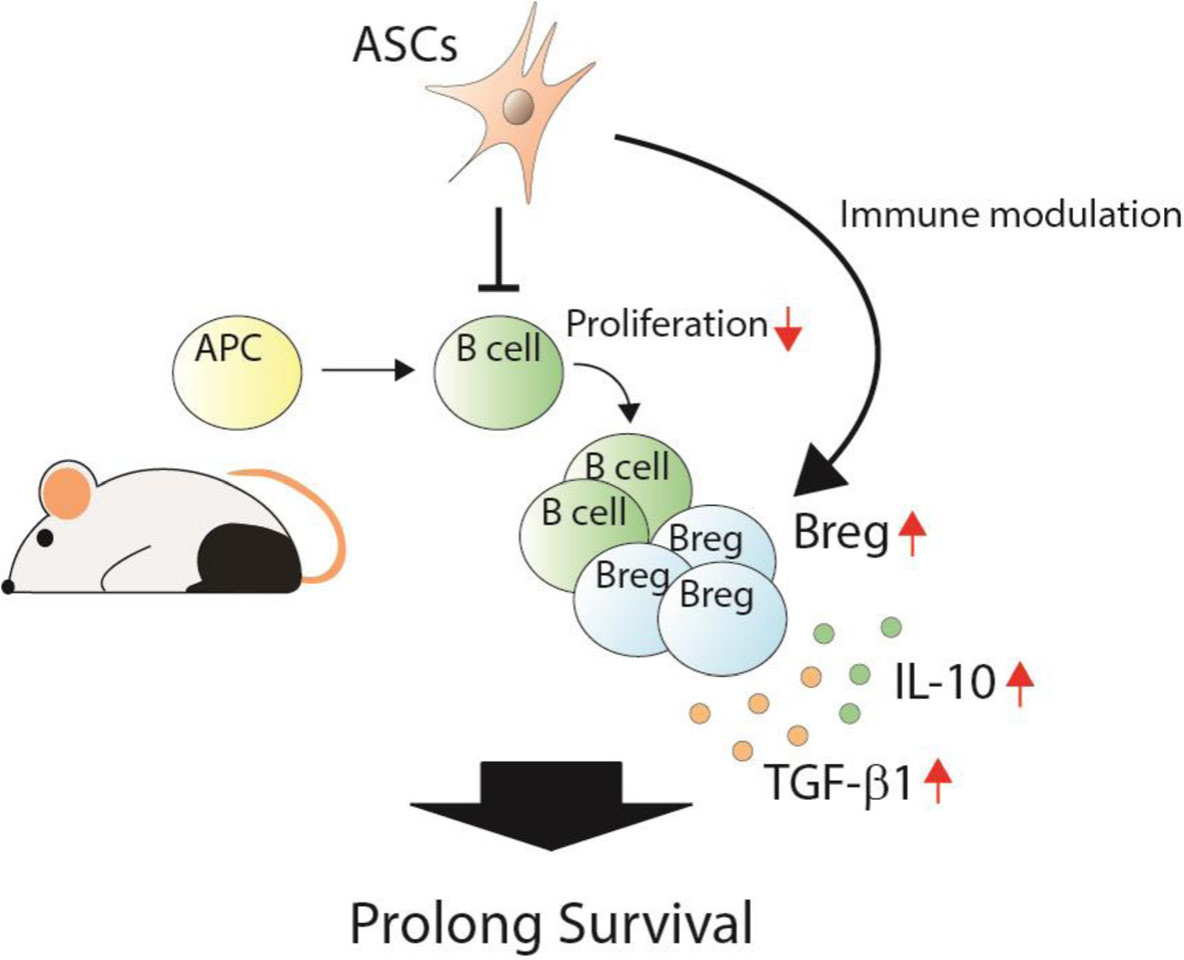
Proposed mechanisms involved in ASC modulation of B cell regulation in allotransplantation. ASCs combined with short-term immunosuppressants prolong allotransplant survival by modulating B cell proliferation, enhancing Breg expression, and modulating the secretion of related cytokines such as IL-10 and TGF-β1, resulting in the prolongation of allotransplant survival and the induction of immune tolerance. ASC, adipose-derived stromal cell; APC, antigen-presenting cell; Breg, regulatory B cell

VCA has been a successful clinical application but not routinely performed because of the consequent requirement of lifelong immunosuppressive agents. Therefore, the development of new strategies to prevent the lifelong use of immunosuppressants or the induction of immune tolerance is awaited. Our previous studies demonstrated that ASCs prolonged allograft survival through modulation of regulatory T cell populations in a rodent hind-limb VCA model. This study demonstrates that ASCs combined with short-term immunosuppressants prolong allograft survival by modulating B cell proliferation, enhancing Breg expression and production of related cytokines, and modulating complement deposition (Fig. 7). The use of ASCs could be a good strategy to induce immune tolerance, and ASCs show potential for future clinical applications for VCA.

## Data Availability

The datasets used and/or analyzed during the current study are available from the corresponding author on reasonable request.

## Abbreviations

ALS: Anti-lymphocyte serum
APC: Antigen-presenting cell
ASCs: Adipose-derived mesenchymal stromal cells
BN: Brown-Norway
BrdU: Bromodeoxyuridine
Bregs: Regulatory B cells
CsA: Cyclosporine A
DSAs: Donor-specific antibodies
ELISA: Enzyme-linked immunosorbent assay
HLAs: Human leukocyte antigens
IFN-γ: Interferon-γ
IHC: Immunohistochemistry
IL-10: Interleukin-10
IL-17F: Interleukin-17F
LEW: Lewis
LPS: Lipopolysaccharide
MSCs: Mesenchymal stromal cells
ND: Not detected
TGF-β1: Transforming growth factor-β1
Tregs: Regulatory T cells
VCA: Vascularized composite allotransplantation
